# Structural Response of the Water Dimer to an Electric Field

**DOI:** 10.1021/acsomega.6c00894

**Published:** 2026-06-18

**Authors:** Bruna Cristina Corcino Carneiro, Fernando Lessa Carneiro, Leonardo Souza Barbosa, David Lima Azevedo

**Affiliations:** † Instituto Federal do Tocantins, 77760-000 Colinas do Tocantins, TO, Brazil; ‡ Instituto de Física, 664468 Universidade de Brasília, 70910-900 Brasília, DF, Brazil; § Departamento de Física, 518709 Universidade Federal do Norte do Tocantins, 77824-838 Araguaína, TO, Brazil; ∥ Programa de Pós-Graduação em Engenharia Aeroespacial (PPgEA), Universidade Estadual do Maranhão, 65055-310 São Luís, MA, Brazil; ⊥ International Center of Physics of Condensed Matter, Instituto de Física, Universidade de Brasília, 70.910-900 Brasilia, DF, Brazil

## Abstract

In this paper, we report simulations of the water dimer under a uniform external electric field carried out within the framework of density functional theory (DFT). By gradually increasing the field intensity, we identify a structural transition characterized by an abrupt change in the orientation of the molecular dipole moments relative to the applied field. Below a critical field strength, the system remains in a conventional water dimer configuration. Above this limit, the dimer undergoes a reorientation, leading to a *cis*-like arrangement associated with a unilateral movement of the donor hydrogen. Vibrational analysis confirms that this field-induced *cis* configuration corresponds to a stable local minimum. We further analyze the structural and electronic properties of this configuration and propose a semiclassical interpretation of the transition based on the interaction between dipole–dipole interactions and the torque exerted by the external electric field within a classical electrodynamic structure. Hysteresis analysis reveals that the electric field drives the system between distinct local minima on the potential-energy surface, stabilizing a high-dipole structure even after the external field is removed.

## Graphical abstract

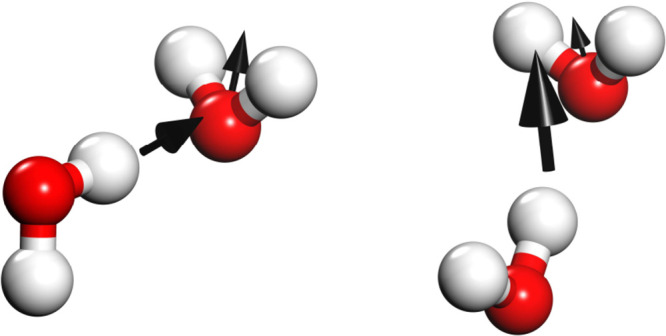

## 1 Introduction

Abundant on our planet, water plays an important role in various physical and chemical processes on Earth,^4–7^ characterized by its polar nature and its predisposition to form hydrogen bonds (H-bonds).^9,10^ Such phenomena arouse interest among researchers dedicated to investigating physical and chemical mechanisms capable of elucidating the anomalous behaviors observed in water.^8,11–13^

In this context, attention turns to intermediate structures, whose molecular organization may offer relevant clues about the origin of such properties. These configurations emerge as potential conceptual links between the microscopic behavior of molecules and the macroscopic properties of the liquid. Molecular clusters are an intermediate between rigid bodies and individual molecules.^14^ This characteristic allows the system to behave as a whole (single molecule) under certain conditions but also to manifest particularities in other interactions, maintaining the cohesion of the cluster.

It is known that water clusters are present in normal water.^15–17^ Their behavior can confer significant properties to many biological and other systems that contain water. Water clusters are represented as (H_2_O)*_n_*, where *n* ≤ 20. Many of the so-called “anomalous behaviors” observed in water are due to the presence of hydrogen bonds, and it is these hydrogen bonds that maintain the cohesion of small water clusters.

Considering the formation of these clusters, it becomes relevant to observe the smallest stable structure derived from these interactions, namely, the water dimer. The simplest water cluster that we can form by using only water molecules is the water dimer. The way this structure is established is pointed out as one of the possible factors associated with the manifestation of properties considered anomalous in the behavior of water.^18^ These dimer structures have also served as test subjects for comparing theoretical and experimental data.^19,20,47^

The implications associated with the presence of water dimers range from conceptual aspects related to the understanding of hydrogen bonding to issues linked to the biological activity of water and atmospheric phenomena on a macroscopic scale.^48,49^ As early as the 1950s, investigations into the effects of infrared radiation on these interactions were conducted using water suspended in solid nitrogen; based on the changes observed in the measured frequencies, it became possible to identify and characterize polymeric species formed in this environment.^50^ Recent studies, both theoretical and experimental, have focused on analyzing the vibrational behavior of hydrogen bonds, allowing the identification of a set of 188 transitions distributed across three distinct vibrational subbands.^51^

An external electric field alters the distribution of electron density. Its application induces the polarization of electrons, atoms, and dipoles, resulting in the eventual reorientation of molecules along the applied electric field. As such, the electric field is one of the important factors capable of modifying the properties of water. Several experimental^1–3,21–15^ and theoretical^9,23–26^ studies have been conducted in an attempt to understand the effects of the electric field on water.

In this work, we employ simulations based on density functional theory (DFT) to investigate the structural and electronic responses of the water dimer to the presence of a uniform external electric field. We start from a stable configuration of the dimer in what we define as the *trans* geometry and analyze its structural evolution as the intensity of the applied electric field is progressively increased. This procedure allows us to identify the critical field strength associated with the structural transition between two stable configurations, which characterizes a field-induced structural transition. The transition is quantified by mapping the angle between the vector connecting the two oxygen atoms and the direction of the electric field, providing a direct geometric criterion for identifying the critical regime. An equivalent criterion can be obtained from the angle between the total electric dipole moment of the system and the external field.

Although the present study focuses on the water dimer as a minimal hydrogen-bonding system, the results indicate that external electric fields can play a relevant role in controlling the structural landscape of larger water clusters and confined water systems. Field-induced structural reorganizations are therefore expected in environments characterized by the presence of strong local electric fields such as interfaces, biological systems, and nanoconfinement regimes. Most interestingly, given that the structure retains its post-transition configuration even after the electric field is turned off, the dimer retains a memory of its interaction with the electric field.

Initially, we performed a detailed characterization of the geometry and electronic properties of the dimer in the absence of a field, with the aim of validating the computational methodology adopted. The theoretical foundations and details of the method employed are presented in Section 2, while Section 3 is dedicated to the analysis of the dimer structures and the comparison of the results obtained with available experimental data. In this same section, we investigate the behavior of the system under the action of a uniform external electric field, varying its intensity, identifying the new configuration resulting from the transition, and evaluating its structural stability. Finally, in Section 5, we discuss the physical implications of the results of the dipole moments obtained in the simulations and the principles of classical electrodynamics, proposing a semiclassical interpretation for the misalignment of the structure in low-intensity electric fields.

## 2 Computational Methods

The structural relaxation calculations performed in this work were conducted using the ab initio method based on DFT,^27,28^ as implemented in the Biovia Materials Studio package,^29^ using the *DMol* ^3^ module.^30,31^ In the simulations, we employed the generalized gradient approximation (GGA), adopting the Perdew–Burke–Ernzerhof (PBE) exchange and correlation functional.^32–34^

The basis set used was the double numerical polarization (DNP) basis set, which is comparable in size to the Gaussian 6–31G­(*d*,*p*) basis set. This basis set includes polarization functions of type *d* for heavy atoms and type *p* for hydrogen atoms,^35,42^ incorporating polarization effects in all hydrogen atoms and, consequently, improving the accuracy of the results obtained. Additionally, the qualitative results were compared with those obtained from the B3LYP hybrid functional^36–39^ in the context of DFT. Additionally, in order to adequately describe noncovalent interactions, such as hydrogen bonds and van der Waals interactions, a DFT-D-type dispersion correction scheme was employed. In the *DMol* ^3^ code, these corrections are implemented based on the formalism proposed by McNellis, also incorporating additional parametrizations developed by Tkatchenko.^43–45^

We use a Fine integration grid to ensure greater accuracy in the calculation of the structures. For the hysteresis calculations, presented in Table 5, a coarse integration grid was applied. The use of this grid allows for a more accurate calculation of the elements of the Hamiltonian matrix, contributing to the stability and reliability of the optimized geometries. The convergence criteria adopted were as follows: a maximum total energy of less than 1.0 × 10^–5^ Ha, a maximum force of less than 2.0 × 10^–3^ Ha/Å, and a maximum atomic displacement of 5.0 × 10^–3^ Å.

The geometric optimizations were considered converged when the maximum force on each atom was less than 10^–3^ a.u. and the total energy variation between successive iterations was less than 10^–6^ Ha. All calculations were performed assuming a singlet electronic ground state. The external electric field was applied uniformly and treated self-consistently in the electronic structure calculations. In addition, semicore DFT (DSPP) pseudopotentials^40^ were employed, which approximate the electrons of the nucleus by means of a single effective potential. Finally, in order to simulate a more realistic environment, all simulations were performed considering solvation in water, with a dielectric constant of ϵ = 78.54 a.u., at a temperature of 0 K.

## 3 Results and Discussion

In this section, we present the main results obtained in this work as well as the discussion associated with them. In Section 3.1, we apply the computational methods described in Section 2 to investigate well-established properties of water dimers, thus validating the reliability of the methodology used in the simulation of these systems. In Section 3.2, a uniform external electric field is applied to a previously optimized water dimer, and from the controlled variation of the field intensity, the new structural configuration, called *cis*, is determined. Finally, in Section 3.3, we analyze the structural stability and other physical properties associated with the configuration obtained after the transition. To verify that the observed structural transition is not an artifact of the incremental (adiabatic) application of the external electric field, we also performed test calculations in which the target field strength was applied directly, without intermediate optimization steps. In all tested cases, the system relaxed to the same *cis* configuration above the critical field, indicating that the transition does not depend on the specific field ramping protocol. The incremental approach was nevertheless adopted throughout this work because it more closely mimics realistic experimental conditions, where the field strength is typically increased continuously, rather than switched on abruptly.

### 3.1 The Water Dimer

Due to intermolecular interactions mediated by hydrogen bonds, water molecules can spontaneously associate to form dimers in the solid, liquid, and gaseous phases. In the gaseous regime, this association is characterized by an equilibrium reaction, given by

$$2H_{2}O(g)↔(H_{2}O{)}_{2}(g)$$ 1

with a formation equilibrium constant equal to 0.0501 bar^–1^ at a temperature of 298.15 K.^57^

Three structurally plausible conformations have been proposed for the water dimer.^50^ The first corresponds to an open, chain-like geometry in which only one hydrogen bond occurs. The second is a cyclic conformation characterized by the participation of each oxygen atom in a hydrogen bond. Finally, there is the bifurcated structure in which a single oxygen atom acts simultaneously as a donor in two hydrogen bonds. These configurations are shown schematically in Figure 1.

**Figure 1:**
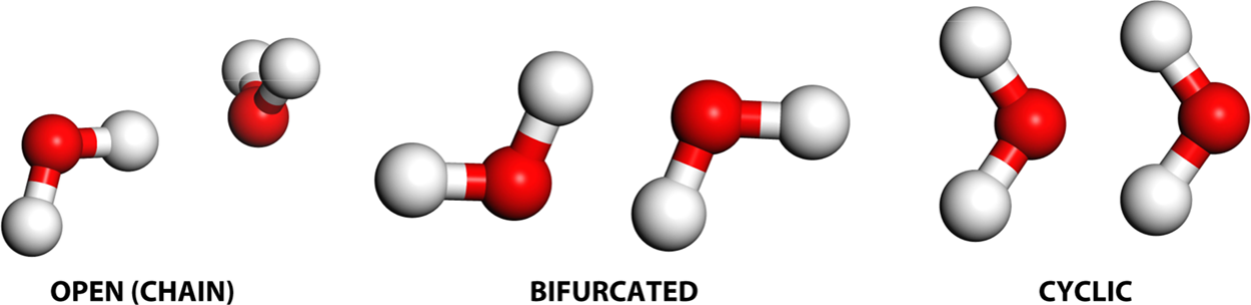
Schematic representation of plausible structural conformations of the water dimer.

In this work, we restrict ourselves to the open, chain-like conformation, which has been the most widely investigated in theoretical studies and appears to be the most consistent in light of recent infrared spectroscopy results.^51^ In this geometry, the two water molecules assume distinct roles as donor and acceptor, with one of the hydrogen atoms participating directly in the interaction in the former, while in the latter, the interaction occurs through an oxygen atom.^52,53^ Throughout the article, the oxygen associated with the donor molecule will be denoted as oxygen 1, while the oxygen of the acceptor molecule will be referred to as oxygen 2. The spatial region between the atoms involved in the hydrogen bond has a significant electronic contribution from the HOMO–4 molecular orbital, corresponding to approximately 40% of the electronic density integral evaluated at the critical point of the bond.^58^

The computational procedures described in Section 2 result in fundamental structural quantities that can be directly compared with experimental data. These quantities include the distance between oxygen atoms 1 and 2, as well as angles Θ_d_ and Θ_a_, defined between the line connecting the two oxygen atoms and the median line formed by the hydrogen atoms of the donor and acceptor molecules, respectively, as illustrated in Figure 2. The scheme shown in Figure 2 corresponds to the geometry of the dimer optimized using the GGA-PBE functional.

**Figure 2:**
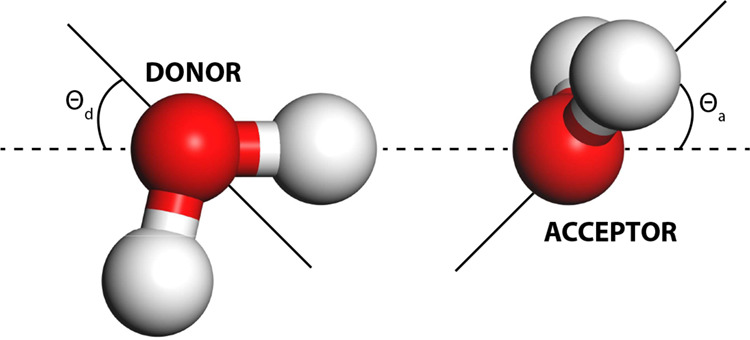
Geometric structure of the water dimer in the open-chain conformation obtained from electronic structure calculations performed with the *DMol* ^3^ code using the PBE exchange–correlation functional.

In this work, the coordinate system was defined to facilitate the description of the geometry and symmetry of the water dimer. The *z*-axis is aligned along the intermolecular O–O vector, while the *yz*-plane is chosen as the reflection plane of the system. Accordingly, the *x*-axis is perpendicular to this plane, such that the transformation *x* → −*x* corresponds to the exchange of the hydrogens of the acceptor molecule. All geometric parameters discussed in this study are expressed within this coordinate framework, and a schematic representation of the adopted coordinate system is provided for clarity. The external electric field is applied along a fixed direction within the *yz*-plane, and its orientation is kept constant throughout the calculations, with only its magnitude being varied.

The values obtained are summarized in Table 1, where they are compared with experimental results reported in the literature.^46,59–63^ It can be observed that both functionals used, the PBE functional and the B3LYP hybrid functional, satisfactorily reproduce the experimental quantities considered, both for the dimer and for the water monomer, respectively.

**Table 1:** Comparison between Experimental and Theoretical Structural Properties of Water Systems in the Absence of an External Electric Field^a^

physical properties	experimental data	PBE	B3LYP
distance O1–O2 (Å)	2.98 ± 0.04 ^59–62^	2.79	2.82
angle Θ_a_ (°)	58 ± 6 ^59–62^	62.10	59.00
angle Θ_d_ (°)	51 ± 6 ^59–62^	51.67	52.02
bond length O–H (Å)	0.9575 ^63^	0.972	0.965
bond angle H–O–H (°)	104.51 ^63^	103.070	103.771

a The table includes geometrical parameters of the water dimer, as well as the bond length and bond angle of the water monomer used for methodological validation. The angles Θ_a_ and Θ_d_ are defined in Figure 2.

The binding energies were calculated for the water dimers in the presence of an external electric field. For each electric-field strength considered, the interaction energy was obtained from the difference between the total energy of the dimer and the sum of the energies of the two isolated water monomers computed at the same level of theory. Thus, the binding energy was defined as

$$E_{\mathrm{binding}}=(E_{\dim\mathrm{er}}-2\times E_{\mathrm{monomer}})$$ 2

It is observed that the presence of the external electric field leads to more negative interaction energies, indicating that the field increases the cohesion between water molecules and stabilizes the dimer structure, as shown in Figure 3. This behavior can be attributed to the alignment of the molecular dipole moments with the external field, consequently increasing the stability of the cluster.^56,72^

**Figure 3:**
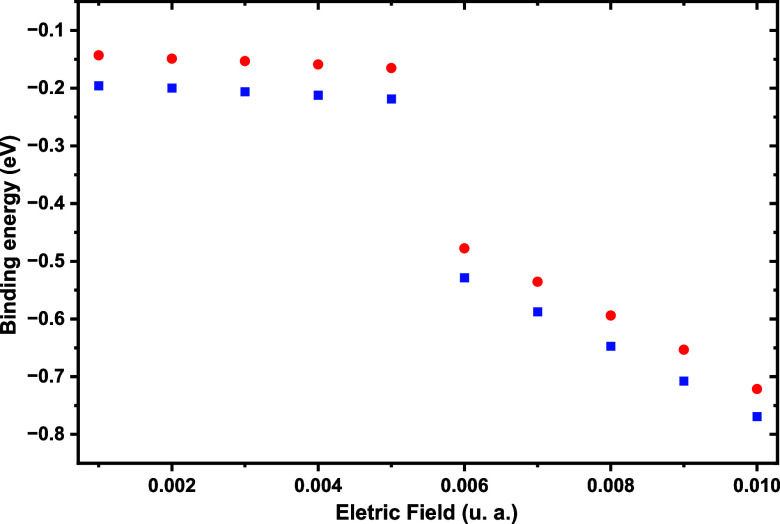
Binding energy (eV) of the water dimer as a function of the applied external electric field (a.u.). For field strengths up to approximately 0.005 a.u., the system remains in the *trans* configuration, while for fields above ∼0.006 a.u., a transition to the *cis* configuration is observed. Results obtained with the B3LYP functional are shown in red, whereas those computed with the PBE functional are shown in blue. This behavior reflects the progressive stabilization of the dimer with increasing field intensity, driven by the alignment of the molecular dipoles with the external field.

### 3.2 Water Dimer under a Uniform Electric Field

During the geometric optimization process, the coordinate system may undergo linear transformations, such as global translations and rotations. As a result, direct identification of the transformations experienced by the structure based on Cartesian coordinates may become nontrivial. Thus, before applying the external electric field, it is convenient to rotate the coordinate system in order to align the vector

$$\vec{R}={\vec{r}}_{O2}-{\vec{r}}_{O1}$$

where ${\vec{r}}_{O1}$ and ${\vec{r}}_{O2}$ denote the positions of the donor and acceptor oxygen atoms, respectively, perpendicular to the direction of the applied electric field. Identifying the direction of the electric field, $\vec{E}$ , with the Cartesian axis, *ĵ*, this condition can be expressed by

$$\vec{R}\cdot \vec{E}=0$$

To circumvent ambiguities associated with residual rotations and translations of the coordinate system, we introduce the angle θ between vectors, $\vec{R}$ and $\vec{E}$ , defined as

$$\theta =\cos^{-1}\left(\frac{\vec{R}\cdot \vec{E}}{\left|\vec{R}\cdot \vec{E}\right|}\right)$$ 3

The angle θ is a scalar quantity independent of the choice of coordinate system. Thus, the analysis based on θ is invariant under global rotations and translations of the system, which do not alter the relative geometry between the vectors, $\vec{R}$ and $\vec{E}$ . By defining the center of charge as the origin of the coordinate system, it is ensured that any rigid displacements of the structure do not introduce ambiguities in the characterization of the molecular orientation relative to the external electric field.

The choice of the angle θ as a fundamental geometric parameter allows a direct comparison with previous results in the literature investigating the effect of external electric fields on the water dimer. Previous studies report the existence of a critical electric field around *E* ≈ 0.008 a.u., in which maxima in the dissociation energy and HOMO–LUMO gap are observed, accompanied by significant changes in the distances and angles associated with the hydrogen bond.^41,73^

When the water dimer is subjected to a uniform external electric field, $\vec{E}=E\hat{j}$ , with $E≡|\vec{E}|$ , no significant alignment of the total dipole moment of the system with the direction of the field is observed for low-intensity fields. Analysis of the individual dipole moments of the constituent molecules reveals that they also do not align with $\vec{E}$ in this regime, resulting in an optimized geometry visually very similar to that shown in Figure 2, both for the GGA-PBE functional and for the B3LYP hybrid functional.

The gradual increase in electric field intensity produces only marginal variations in angle θ until a certain threshold is reached. To investigate this behavior, the field was applied incrementally: starting at *E* = 1 × 10^–3^ a.u., the structure was optimized, and then the field was successively increased in increments of 1 × 10^–3^ a.u., continuing this procedure until more intense fields were reached. In the range 5 × 10^–3^ a.u. ≤ *E* ≤ 6 × 10^–3^ a.u., a qualitative change in the behavior of the system is observed. In this regime, the dimer begins to show partial alignment with the external electric field, evidencing a more pronounced geometric reorganization. This effect is illustrated in Figure 4a for the GGA-PBE functional. The complete alignment of the system with the field would correspond to the limit θ = 0, which is not reached in the range of fields considered.

**Figure 4:**
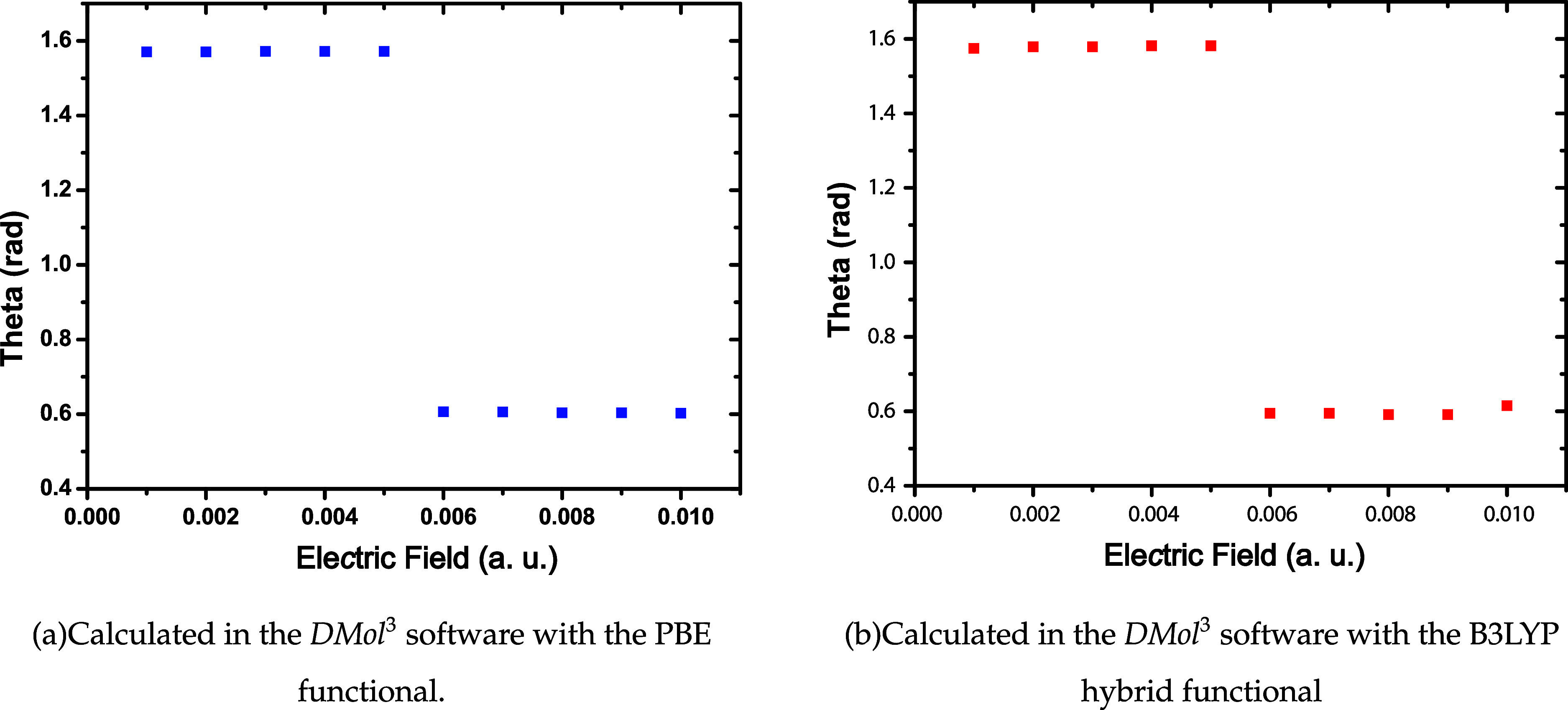
Angle θ between the vectors, $\vec{R}$ and $\vec{E}$ , as a function of the applied electric-field intensity *E* obtained from the simulations.

We observe that sharp variations of θ in fields in the magnitude order of 0.008 a.u. are compatible with what has been proposed in recent studies,^41^ in which the decrease in the O–H–O angle and the growth of the dipole moment associated with variations in the length of the hydrogen bond act cooperatively to reduce the total energy of the system. More intense fields may therefore favor structurally more elongated configurations, constituting a precursor stage to dimer dissociation.

Qualitatively similar behavior is observed in Figure 4b for the B3LYP hybrid functional. In particular, from Figure 4, a discontinuous jump in the values of θ can be identified when the electric field is increased from 5 × 10^–3^ to 6 × 10^–3^ a.u. in the case of the GGA-PBE functional. It can be noted that the orientation of the structure changes very little as the field strength increases further. The discrete behavior observed is characterized as an intrinsically quantum manifestation of the system. In contrast, the partial misalignment of the dimer in weak electric fields can be understood from a semiclassically motivated approach, as discussed in Section 5. The emergence of a new range of values for the angle θ after exceeding the critical field indicates the formation of a structural configuration distinct from the previous one, thus corresponding to a new geometric isomer of the dimer. We refer to the initial configuration as *trans*, while the structure resulting from the transition induced by the electric field is designated as *cis*. The results show that the geometric changes induced by the electric field persist in the gas phase, and the *cis* geometry remains stable under these conditions. The stability and electronic and structural properties associated with this new configuration are analyzed in the following subsection.

### 3.3 Water Dimer *Cis* Configuration

In Section 3.2, we show that the water dimer initially in the *trans* configuration undergoes a structural reorganization to the *cis* configuration when subjected to a uniform external electric field. For the PBE functional, the structure remains in the *trans* configuration for electric fields of magnitude equal to or less than *E* = 0.005 a.u., while for field intensities equal to or greater than *E* = 0.006 a.u., the dimer relaxes to the *cis* configuration. The two distinct geometries are compared in Figure 5a,b, which also shows the total electric dipole moment of the dimer as well as the individual dipole moments associated with each water molecule.

**Figure 5:**
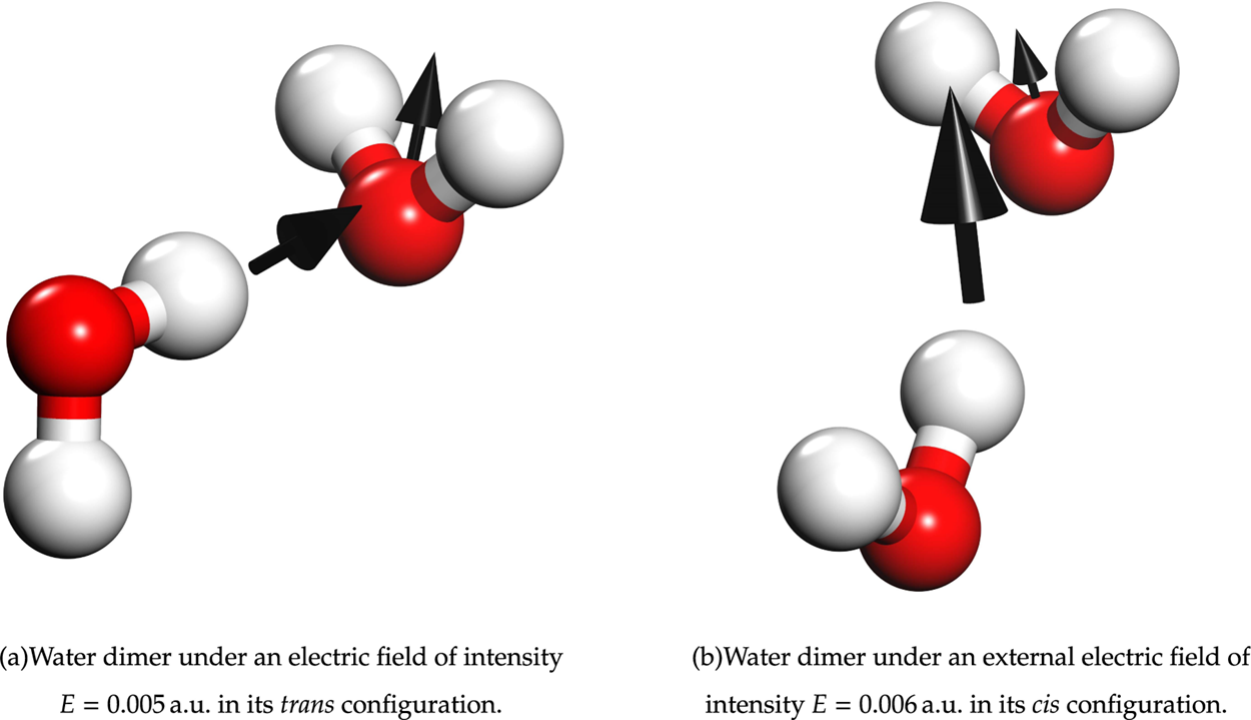
Water dimer subjected to an external electric field, simulated using the *DMol* ^3^ software with the B3LYP hybrid functional. Arrows denote the dipole moments associated with each molecule.

The transition appears to occur with the donor hydrogen undergoing a wag, similar to intermolecular vibrations due to its interaction with radiation.^51^ The new *cis* configuration is achieved with the aid of the electric field but does not need the electric field to remain in the new configuration, i.e., by turning off the field and performing geometric optimization, the structure remains in the *cis* configuration. This latter fact suggests that the structure in the new configuration is stable without the field with the former acting as a catalyst. The issue of the stability of the *cis*configuration is addressed at the end of this subsection.

Some physical properties, analogous to those shown in Table 1, of the *cis* water dimer are summarized in Table 2. The angles Θ_a_ and Θ_d_ can be seen in Figure 6. The properties are obtained from the new configuration without the electric field, i.e., the field is turned off and a geometric optimization is performed on the already transitioned structure.

**Table 2:** Properties of the *Cis* Dimer^a^

physical properties	PBE	B3LYP
distance O1–O2 (Å)	2.925	2.929
angle Θ_a_ (°)	61.231	57.190
angle Θ_d_ (°)	127.498	126.963

a The angles are shown in Figure 6.

**Figure 6:**
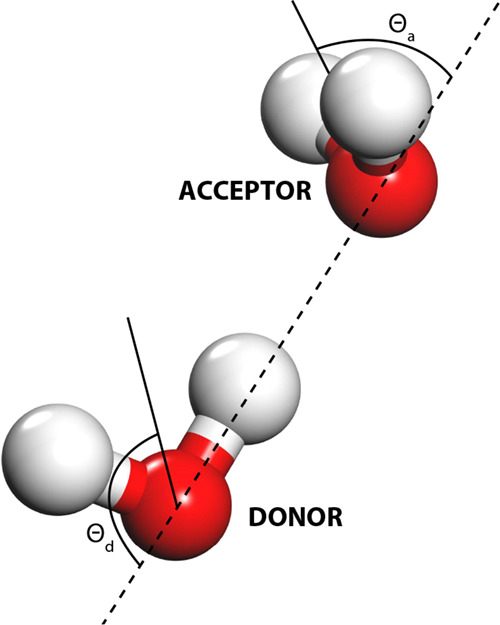
Water dimer in the *cis* configuration, simulated in the *DMol* ^3^ software with the PBE and the B3LYP hybrid functional, is presented in the same reference system adopted for the *trans* structure. The rotation observed around the *ĵ*-axis does not correspond to an arbitrary rotation of the coordinate system but results from the structural reorganization of the cluster itself induced by the application of the external electric field.

The highest occupied molecular orbital (HOMO) and lowest unoccupied molecular orbital (LUMO) energies of the neutral *trans* and *cis* configurations are presented in Table 3 for comparison. The corresponding HOMO–LUMO gaps are also reported. As can be seen, both configurations exhibit very similar gaps (9.989 eV for *trans* and 9.972 eV for *cis* at the B3LYP level). These values are smaller than the experimentally estimated gap of (11.16 ± 0.05) eV reported in ref 64, which is consistent with the well-known tendency of conventional DFT approaches to underestimate electronic energy gaps. The results obtained with the PBE functional show a further underestimation, as expected for semilocal functionals.

**Table 3:** Frontier Orbitals HOMO and LUMO Calculated for the *Trans* and *Cis* Dimer Configurations^a^

	*trans*		*cis*	
	PBE	B3LYP	PBE	B3LYP
HOMO (eV)	–6.584	–8.236	–6.597	–8.195
LUMO (eV)	1.084	1.753	1.053	1.712
energy gap (eV)	7.671	9.989	7.650	9.972

a Energy gap is the HOMO–LUMO gap in eV.

The stability of the new configuration can be evaluated by analyzing its vibrational frequencies, which are calculated from the Cartesian matrix of the second derivatives of the energy with respect to the nuclear coordinates, known as the Hessian matrix of the molecular (or periodic) system.

A molecular vibration occurs when the molecule absorbs a quantum of energy, with the fundamental vibration being excited when this absorption occurs in the ground state. In the context of harmonic vibrational analysis, frequencies associated with negative eigenvalues of the Hessian matrix manifest as imaginary values and indicate directions of the potential-energy surface in which the energy does not correspond to a local minimum, generally characterizing the transition states. The normal mode associated with this eigenvalue describes the nuclear displacement that drives the system toward a local energy minimum.

Thus, the evaluation of vibrational frequencies is a robust criterion for determining the structural stability of molecules or periodic systems: the exclusive presence of real and positive frequencies indicates that the structure corresponds to a local minimum of the potential-energy surface, that is, to a stable and nontransient configuration. The obtained vibrational frequencies are shown in Figure 7b for the *trans* configuration (in the absence of an electric field and before the structural transition) and in Figure 7 for the configuration resulting after the transition, also in the absence of an electric field.

**Figure 7:**
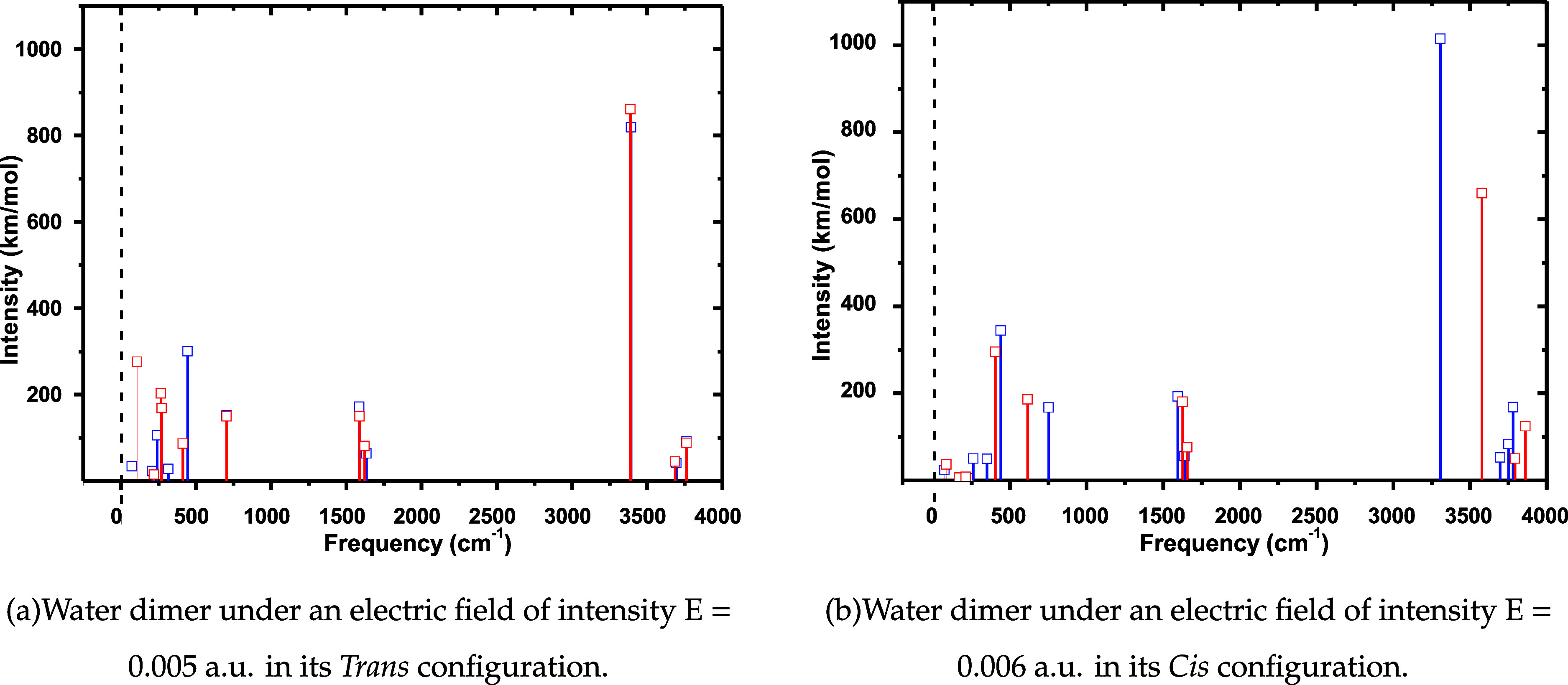
Vibrational frequencies of the water dimer configuration calculated using the *DMol* ^3^ software with the PBE functional (red squares) and the B3LYP hybrid functional (blue squares).

From Figure 7, it can be seen that both the PBE functional and the B3LYP hybrid functional, using the DNP basis set, produce only real and positive vibrational frequencies, which indicates that the structure resulting from the field-induced transition corresponds to a local minimum of the potential-energy surface and is therefore structurally stable. The fact that the configuration obtained in the presence of an external electric field remains stable even after the field is removed shows that electric fields can be used as a control mechanism to obtain different stable structures of water dimers.

As discussed in ref 51, the water dimer has six intermolecular vibrational modes, wag with donor torsion, acceptor torsion, acceptor oscillation, O–O stretching, in-plane bending, and out-of-plane bending. Our results suggest that the electric field acts particularly effectively on the modes associated with the donor hydrogen, promoting a persistent oscillation that favors the structural rearrangement observed during the transition.

Additionally, it is observed that the total energy of the *cis* configuration is lower than that of the *trans* configuration for both functionals considered, as shown in Table 4, corroborating the greater energy stability of the *ci* *s* phase in the post-transition regime.

**Table 4:** Water Dimer Total Energies in the Absence of an Electric Field for the *Trans* Configuration and the *Cis* Configuration

water dimer total energies (eV)		
	*trans*	*cis*
PBE	–4157.533	–4157.535
B3LYP	–4380.331	–4380.334

### 3.4 Hysteretic Response and Metastability

The structural transition discussed above raises an important question: whether the electric field only produces a continuous and reversible deformation of the water dimer or whether it drives the system from one structural basin to another. In order to distinguish between these two possibilities, we performed an up-and-down electric-field cycle. In this protocol, the geometry optimized at a given value of the electric field was used as the initial geometry for the next value of the field; thus, the calculation preserves the structural history of the system along the cycle.

The upward scan was initiated from the optimized zero-field structure and the field was increased step by step. After the high-field structure was obtained, the field was decreased back to zero, again using the optimized geometry of the previous step as the input geometry for the next one. The cycle may be written schematically as

$$0\to E_{\max}\to 0$$ 4

This procedure is particularly useful because a reversible deformation should lead to nearly coincident upward and downward branches. In contrast, if the system switches between two metastable basins, the downward path need not retrace the upward one.

Figure 8 shows the result of this field cycle using the same numerical integration setting employed in the main calculations of this work. The most direct signature of the transition is observed in the dipole component along the field direction, μ_y_. During the upward scan, the system remains in a low-dipole branch up to *E* = 0.004 a.u. Between *E* = 0.004 and *E* = 0.005 a.u., μ_*y*_ increases abruptly, indicating a sudden transition to a high-dipole branch. Once this high-dipole branch is reached, the downward scan does not return to the initial low-dipole branch. Instead, the system remains in the high-dipole branch down to zero field.

**Figure 8:**
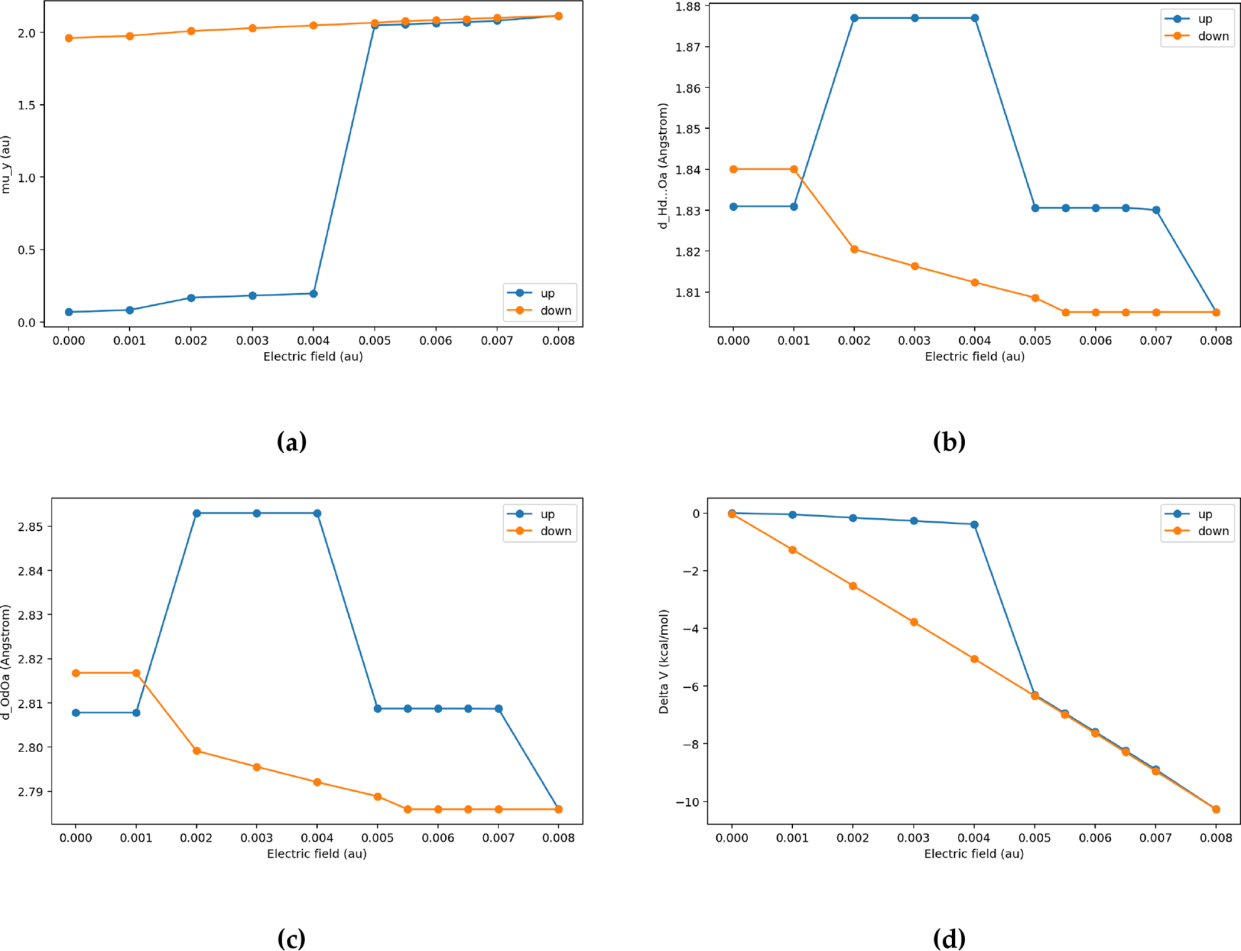
Up-and-down electric-field cycle obtained with the same numerical integration setting used in the main calculations. The upward and downward branches are shown for the (a) dipole component along the field direction, μ_*y*_; (b) hydrogen-bond distance, *d*_H_d_···O_a__; (c) oxygen–oxygen distance, *d*_O_d_O_a__; and (d) relative energy, Δ*V*. During the upward scan, the system remains in a low-dipole branch up to *E* = 0.004 a.u. and then jumps to a high-dipole branch between *E* = 0.004 a.u. and *E* = 0.005 a.u. In the downward scan, the system remains in the high-dipole branch down to zero field. This path dependence characterizes a hysteretic structural transition.

The same path dependence is also observed in the intermolecular distances. Both the hydrogen-bond distance *d*_H_d_···O_a__ and the oxygen–oxygen distance *d*_O_d_O_a__ display discontinuous changes in the same field interval where the dipole jump occurs. This shows that the hysteresis is not merely an electronic polarization effect but is accompanied by a genuine structural reorganization of the dimer. The energy profile is also consistent with this interpretation: after the system reaches the high-dipole branch, the electric field stabilizes this branch more strongly, as expected from the leading field-coupling contribution to the energy,

$$V(E)≃V_{0}-\mu_{y}E$$ 5

where *V* (*E*) is the energy for the electric field *E*, *V*_0_ is the energy without the presence of the electric field, and μ_*y*_ is the electric dipole not along the *y* direction. Therefore, the field-induced transformation is not simply a reversible distortion of the initial structure. Rather, the external field drives the water dimer from an initial low-dipole basin to a distinct high-dipole basin. The persistence of the high-dipole structure after the field is removed is the signature of hysteresis and indicates that the two structures are separated by an effective barrier on the potential-energy surface.

The angular coordinate, θ_d_, although useful for monitoring the local orientation of the donor hydrogen, is not the most sensitive order parameter for this transition. In the present cycle, the most pronounced signatures of the basin switch are the dipole component, μ_*y*_, and the intermolecular distances. This explains why a constrained scan along θ_d_ alone does not fully characterize the transition pathway: the relevant structural reorganization involves a collective change of the dimer geometry and its polarization response.

To verify that the initial and final zero-field structures correspond to genuine local minima, we performed vibrational-frequency calculations for the two end points of the cycle. The initial end point is the zero-field structure before the application of the field, while the final end point is the structure obtained after increasing the field, inducing the transition, and reducing the field back to zero. The calculation was carried out for the numerical integration setting used in the main calculations and was also repeated with a finer integration setting.

The results are summarized in Table 5. In all cases, no imaginary frequencies were found. Therefore, both the initial low-dipole structure and the final high-dipole structure are local minima on the zero-field potential-energy surface. This confirms that the field-induced cycle does not produce a transient distortion. Instead, it connects two metastable basins of the water dimers.

**Table 5:** Zero-Field Vibrational Stability of the Initial and Final Structures Obtained from the Electric-Field Cycle^a^

protocol	branch	*N*_imag_	lowest positive frequency (cm^–1^)	Δ*E* (kcal/mol)
coarse	up	0	178.9	0.000
coarse	down	0	199.0	–0.031
fine	up	0	69.0	0.000
fine	down	0	170.8	–0.060

a The relative energy is measured with respect to the corresponding initial zero-field structure of each numerical protocol.

The relative energies of the two zero-field end points are very close, but the final high-dipole structures are slightly lower in energy within both numerical protocols. We do not attach significance to the small numerical differences themselves. The relevant point is that the final structure remains as a stable local minimum after the field is removed. Together with the hysteretic field cycle, this demonstrates that the field transfers the dimer from one zero-field basin to another.

We also repeated the up-and-down cycle using a finer numerical integration setting. The apparent transition interval is shifted with respect to the calculation shown in Figure 8, indicating that the precise numerical value of the apparent threshold field depends on the computational protocol. This sensitivity is expected for geometry optimization near a basin-switching threshold. However, the qualitative result is unchanged: the system undergoes a field-induced transition to a high-dipole branch and remains in that branch when the field is reduced to zero. Thus, the robust physical conclusion is not the exact numerical value of the apparent transition field but the existence of a hysteretic structural transition between two metastable basins.

This analysis provides a physical interpretation for the persistence of the field-induced structure after the external field is removed. The electric field does not simply deform the original *trans*-like configuration. Rather, it drives the dimer across an effective barrier into another local minimum on the potential-energy surface. Once this new basin is reached, removal of the field does not restore the original geometry, giving rise to the observed structural memory.

## 4 Electric Dipole Moment Analysis

The semiclassical dipole–dipole model employed here should be regarded as a heuristic tool rather than a quantitative description of the system. Its purpose is not to reproduce the full energetic landscape obtained from first-principles calculations but to provide physical intuition regarding the tendency of the water molecules to align with the external electric field. While this simplified model correctly captures the progressive alignment of molecular dipoles, it fails to account for electronic redistribution and geometric relaxation effects, which are essential to explain the stabilization of the *cis* configuration at high field strengths. Therefore, the field-induced structural transition discussed in this work emerges from intrinsically quantum-mechanical effects that go beyond the scope of a purely classical electrostatic picture. The response of the water dimer to an external electric field can be better understood by analyzing its electric dipole moments. In the *trans* configuration, the total dipole moment of the cluster is less aligned with the applied electric field than in the *cis* configuration, as illustrated in Figure 9, where perfect alignment corresponds to a zero-degree angle. This behavior hints at the nonrigid nature of the water dimer, in which individual molecules retain a certain degree of independence in their interaction with the field.

**Figure 9:**
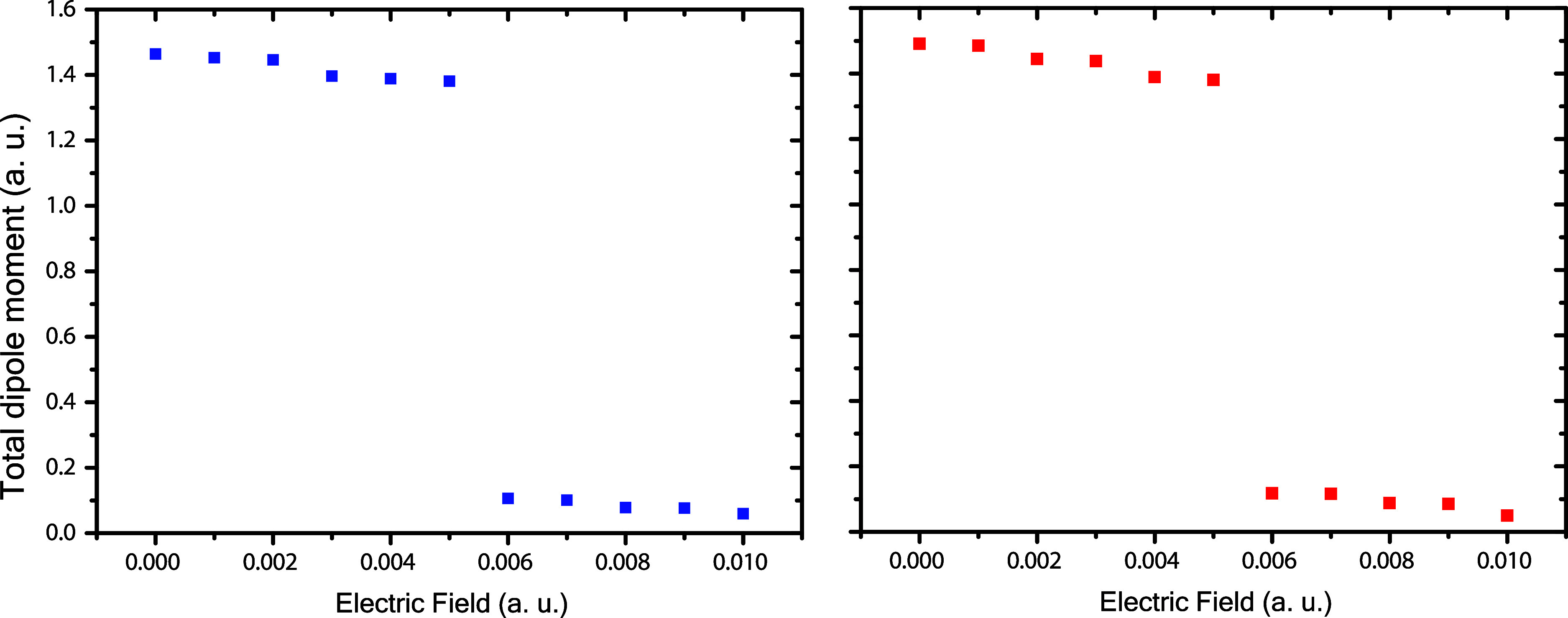
Total electric dipole moment of the water dimer as a function of the applied electric field. Results obtained using the PBE functional are shown on the left-hand side, while those obtained with the B3LYP hybrid functional are shown on the right-hand side.

Each water molecule has its own electric dipole moment, which interacts not only with the external electric field but also with the dipole moments of the neighboring molecules. At lower field intensities, the interaction between individual molecular dipoles prevents the total dipole moment of the cluster from being aligned with the field.

However, when the electric field becomes sufficiently strong, the cluster undergoes a structural reorganization that allows for a greater degree of alignment, consistent with the existence of field-dependent response regimes reported in the literature.^54^

In the *trans* configuration, the total dipole moment of the cluster is less aligned with the applied electric field than that in the *cis* configuration, as illustrated in Figure 9, where perfect alignment corresponds to a zero-degree angle. This behavior highlights the nonrigid nature of the water dimer, in which individual molecules retain a certain degree of independence in their interaction with the field.

Each water molecule has its own electric dipole moment, which interacts not only with the external electric field but also with the dipole moments of the neighboring molecules. At lower field intensities, the interaction between individual molecular dipoles prevents the total dipole moment of the cluster from being aligned with the field.

However, when the electric field becomes sufficiently strong, the cluster undergoes a structural reorganization that allows for a greater degree of alignment, consistent with the existence of field-dependent response regimes reported in the literature.^54^

In this sense, the lack of alignment under weaker fields can be interpreted as the donor oxygen acting as a potential barrier that prevents the hydrogen atom from reaching the *cis*configuration. Once the field exceeds an intensity threshold, this barrier can be overcome, leading to the observed structural transition.

Within this classical structure, the electric field exerts a torque on each molecular dipole, which tends to align it with the direction of the field. Simultaneously, the dipole–dipole interaction generates additional torques that can oppose this alignment.

It is important to note that the classical dipole model here does not attempt to describe the microscopic mechanism of the structural transition itself, which is profoundly quantum mechanical. Instead, it provides a qualitative interpretation of the system’s response to the electric field, consistent with modern analyses that describe the dipole moment of water as an emergent quantity associated with environment-induced electronic redistribution.^55^ Instead, this model provides a qualitative view of the lack of alignment of molecular dipoles under weak and moderate electric fields, prior to structural rearrangement.

For the *trans* configuration, the resulting balance between these competing torques leads to a net effect that disfavors alignment at lower field intensities. As the electric field strength increases, the torque associated with the field increases, while the dipole–dipole interaction remains approximately constant. Consequently, a field strength threshold beyond which the alignment becomes favorable is expected.

From a classical electrodynamics perspective, the behavior of the *trans* configuration under an external electric field may be interpreted in terms of competing torques acting on the molecular dipoles. For the *trans* configuration, the torques produced by the interaction of dipoles 1 and 2 with the electric field (in units of 10^–21^Nm) are

$${\vec{\tau }}_{1t}(E=0.005\;a.u.)=10.275x+56.854z$$ 6

$${\vec{\tau }}_{2t}(E=0.005\;a.u.)=2.202x+14.427z$$ 7

Meanwhile, the torque exerted by dipole 2 on dipole 1 (in units of 10^–19^ N m) is

$${\vec{\tau }}_{21t}(E=0.005\;a.u.)=-{\vec{\tau }}_{12t}(E=0.005\;a.u.)=-12.890x+5.356y-65.529z$$ 8

The torques ${\vec{\tau }}_{12t}$ and ${\vec{\tau }}_{21t}$ arise from the dipole–dipole interaction and are present even in the absence of an external field, whereas ${\vec{\tau }}_{1t}$ and ${\vec{\tau }}_{2t}$ originate from the interaction between the dipoles and the electric field, respectively.

The electric field tends to rotate the dipole ${\vec{\mu }}_{1t}$ in order to align it with the field. However, the interaction with the second dipole opposes this rotation. This can be seen by evaluating the total torque acting on dipole 1,

$$\sum {\vec{\tau }}_{1}={\vec{\tau }}_{1t}+{\vec{\tau }}_{21t}\approx {\vec{\tau }}_{21t}$$

Therefore, for weak electric fields, the dipole–dipole interaction dominates over the field–dipole interaction, preventing the alignment of the cluster.

As the electric field increases, the alignment torque ${\vec{\tau }}_{1t}\propto \vec{\mu }\times \vec{E}$ grows linearly with *E*, whereas the dipole–dipole torque ${\vec{\tau }}_{21t}$ remains approximately constant. Consequently, a threshold field is expected where the alignment torque becomes comparable to the dipole–dipole torque.

Our calculations show that this occurs close to *E* ≈ 0.006 a.u., where the system approaches a quasi-aligned configuration. However, instead of a rigid alignment of the *trans* structure, the cluster undergoes a structural reconfiguration leading to the *cis* configuration.

This indicates that while classical electrodynamics explains the resistance of the cluster to alignment under weak fields, the structural rearrangement itself cannot be described purely within a classical framework. The transition likely involves quantum nuclear effects associated with hydrogen-bond rearrangement, which are known to play an important role in water clusters.^67–70^

It is important to note that the alignment observed at higher field intensities is not a simple rotation of the original *trans* configuration. Instead, it is accompanied by a structural reorganization of the cluster, resulting in the formation of the *cis* configuration. Although classical electrodynamics offers an explanation for nonalignment under weak fields, it does not account for the structural transition itself, which is inherently quantum mechanical. Tunneling effects, known to occur in hydrogen-bridged water systems,^67–70^ may play a role in facilitating this transition.

In the *cis* configuration, the molecular dipole moments are more aligned with each other, as illustrated in Figure 5. From a classical point of view, such an arrangement corresponds to a higher dipole–dipole interaction energy. The interaction energy between two electric dipoles ${\vec{\mu }}_{1}$ and ${\vec{\mu }}_{2}$ separated by a distance *r* is given by^71^

$$U_{12}=\alpha \left[\frac{1}{r^{3}}({\vec{\mu }}_{1}\cdot {\vec{\mu }}_{2})-\frac{3}{r^{5}}({\vec{\mu }}_{1}\cdot \hat{r})({\vec{\mu }}_{2}\cdot \hat{r})\right]$$ 9

where α is a constant that depends on the unit system and *r̂* is the unit vector connecting the two dipoles. Using the dipole moments reported in Table 6, the semiclassical interaction energies were evaluated for both configurations.

**Table 6:** Electric Dipole Moments of the Individual Water Molecules in the *Trans* and *Cis* Configurations^a^

water dimer electric dipole moments (a.u.)			
	*x*	*y*	*z*
${\vec{\mu }}_{1t}$	2.608	0.510	–0.471
${\vec{\mu }}_{2t}$	0.662	1.589	–0.101
${\vec{\mu }}_{1c}$	–0.447	4.215	–0.034
${\vec{\mu }}_{2c}$	–0.558	1.631	0.028

a Calculated using the PBE functional.

The interaction energy for the *trans* configuration is *U*_12*t*_ = −9.064 eV, while for the *cis* configuration, it is *U*_12*c*_ = −3.846 eV. From a purely classical perspective, the *trans* configuration would therefore be energetically favored. The persistence of the *cis* structure after removal of the electric field suggests the presence of a potential barrier that prevents the system from reverting to the *trans* configuration. This barrier likely originates from the same mechanism that inhibits alignment under weak electric fields, indicating that external energy would be required to restore the original structure.

It is important to emphasize that the large energy disparity predicted by the simplified dipole–dipole model (on the order of 6 eV) should not be interpreted as a quantitative estimate of the relative stability between the *cis* and *trans* configurations. The objective of introducing the semiclassical dipole–dipole model, therefore, was not to reproduce the energy obtained from ab initio calculations, but rather to provide a qualitative and intuitive physical framework for understanding how molecular dipoles tend to reorient themselves under the influence of an external electric field. From this perspective, the model serves as a heuristic tool to illustrate the cluster’s tendency to align with the applied field, complementing the more precise description of the DFT. Final comments on the results obtained in this article are provided in the final section below.

## 5 Conclusions

In this work, we investigated the structural response of the water dimer under a uniform external electric field. The results show that the applied field can induce a structural transition between two locally stable configurations, identified as *trans* and *cis*. The transition occurs for electric fields on the order of $O(E)=10^{-3}\;a.u.\approx 10^{8}\;V/m$ , which lies within a physically realistic range, considering that fields on the order of $O(E)=10^{9}\;V/m$ have been experimentally reported.^65^ The *cis* configuration obtained after the field-induced transition remains stable even after removal of the external field. Vibrational-frequency analysis shows that all calculated frequencies are positive (Figure 7), confirming that this structure corresponds to a local minimum on the potential-energy surface. Dipole moment analysis indicates that the *cis* configuration exhibits stronger alignment with the external electric field than the *trans* configuration (Figure 9). These results demonstrate that the electric field can act as an effective control parameter for inducing structural rearrangements in hydrogen-bonded systems. Given that larger water clusters present multiple structural minima,^66^ the present findings suggest that external electric fields may also influence structural stability and transitions in more complex hydrogen-bonded networks. The hysteresis analysis also clarifies why the field-induced structure remains stable after the external field is removed. In an up-and-down electric-field cycle, the system does not retrace the same structural path, but it remains in a high-dipole branch after the field is reduced to zero. Together with the absence of imaginary frequencies for both zero-field end points, this result indicates that the electric field drives the dimer from one metastable basin to another on the potential-energy surface. Therefore, although the precise apparent transition field depends on the computational protocol, the basin-switching character of the transition is a strong qualitative feature.
